# 2-Bromo-6-hydrazinyl­pyridine

**DOI:** 10.1107/S2414314623001694

**Published:** 2023-02-28

**Authors:** Valeri V. Mossine, Steven P. Kelley, Thomas P. Mawhinney

**Affiliations:** a Department of Biochemistry, University of Missouri, Columbia, MO 65211, USA; b Department of Chemistry, University of Missouri, Columbia, MO 65211, USA; University of Aberdeen, United Kingdom

**Keywords:** crystal structure, aryl­hydrazine, hydrogen bonding, halogen bond, π-stacking

## Abstract

The title compound, C_5_H_6_BrN_3_, crystallizes in the ortho­rhom­bic space group *P*2_1_2_1_2_1_ with two mol­ecules with different conformations in the asymmetric unit. In the crystal, N—H⋯N and bifurcated N—H⋯(N,N) hydrogen bonds link the mol­ecules into [100] chains; a short Br⋯Br halogen bond and π–π stacking inter­actions are also observed.

## Abstract 2

The title mol­ecule is essentially flat. In the crystal the mol­ecules are linked by a system of N—H⋯N hydrogen bonds formed by the hydrazinyl group, a Br⋯Br halogen bond, and π-stacking between the pyridine rings.

## Graphical abstract

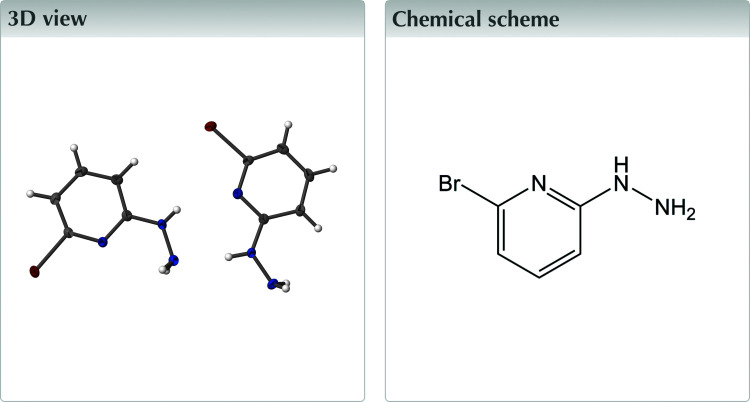

## Structure description

Since Emil Fischer’s discovery of phenyl­hydrazine nearly 150 years ago (Kauffman & Ciula, 1977 ▸), there has been a persistent inter­est in aryl­hydrazines because of their numerous applications in organic chemistry, for instance, as synthetic precursors to a number of anti­microbial (Rollas & Küçükgüzel, 2007 ▸), thrombopoietic (Kuter, 2010 ▸), anti-inflammatory (Fraga & Barreiro, 2006 ▸) or vasodilatory (Reece, 1981 ▸) drugs, but also due to their presence in wild and cultivated mushrooms, with a history of neurotoxic and carcinogenic effects (Toth, 2000 ▸). In the course of our search for inhibitors of bacterial virulence factors (Mossine *et al.*, 2016 ▸, 2020 ▸), we prepared the title compound, which was considered a potential precursor for pharmacologically active, metal-binding hydrazones. Here we report its crystal structure.

**Figure 1:**
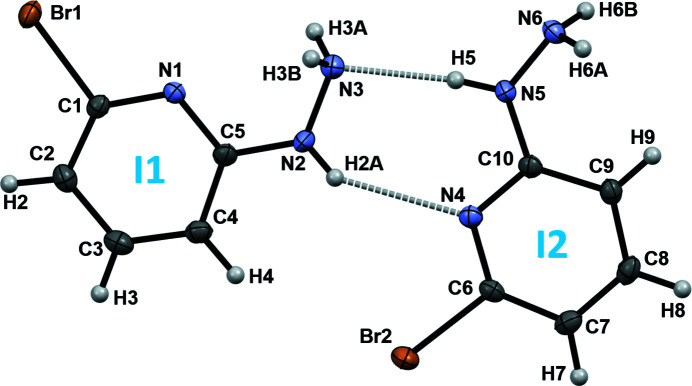
Atomic numbering and displacement ellipsoids at the 50% probability level for mol­ecules (**I1**) and (**I2**). Hydrogen bonds are shown as dashed lines.

The title compound, (**I**), crystallizes in the ortho­rhom­bic space group *P*2_1_2_1_2_1_, with eight mol­ecules per unit cell. The asymmetric unit contains two conformationally non-equivalent mol­ecules of 6-bromo­pyridin-2-ylhydrazine, (**I1**) and (**I2**), as shown in Fig. 1 ▸. All bond lengths and angles are within their expected ranges. The mol­ecules are essentially flat, with the greatest deviations from the average mol­ecular planes, among the non-hydrogen atoms, found for N2 at 0.081 (2) Å and N5 at 0.073 (2) Å in (**I1**) and (**I2**), respectively. The spatial arrangements of the hydrazino groups, as defined by the torsion angles H2*A*—N2—N3—H3*A* = 137 (3)° and H5—N5—N6—H6*A* = 121 (3)°, correspond to the low-energy conformation that has been calculated for acyl hydrazides (Centore *et al.*, 2010 ▸). There is a notable difference between the conformations of (**I1**) and (**I2**), however. While in (**I1**) the hydrazine nitro­gen atom N3 is in the *syn*-disposition with respect to the pyridine nitro­gen atom N1, with N1—C5—N2—N3 = 5.4 (3)°, in (**I2**) the hydrazine group is in the *anti*-conformation, with the corresponding torsion angle N4—C10—N5—N6 = 171.0 (2)°. For comparison, in 3-chloro­pyrid-2-ylhydrazine (Wang *et al.*, 2010 ▸), the hydrazine group is in the *syn*-conformation, with the respective torsion angle being −9.6°. The only other structural analogue of (**I**) for which X-ray diffraction data are available is 2-hydrazino­pyridine; however, no crystal structure of this mol­ecule as a free base is known. In crystalline palladium(II) (Drożdżewski *et al.*, 2006 ▸) and copper(I) (Healy *et al.* 1988 ▸) complexes of 2-hydrazino­pyridine, both the terminal hydrazine and pyridine nitro­gen atoms are co-ordinated to the same metal ion, thus stabilizing the *syn*-conformation of this ligand. In the 2-hydrazino­pyridine di­hydro­chloride salt (Zora *et al.*, 2006 ▸), both the terminal hydrazine and pyridine nitro­gen atoms are protonated and thus forced into the *anti*-conformation.

**Figure 2:**
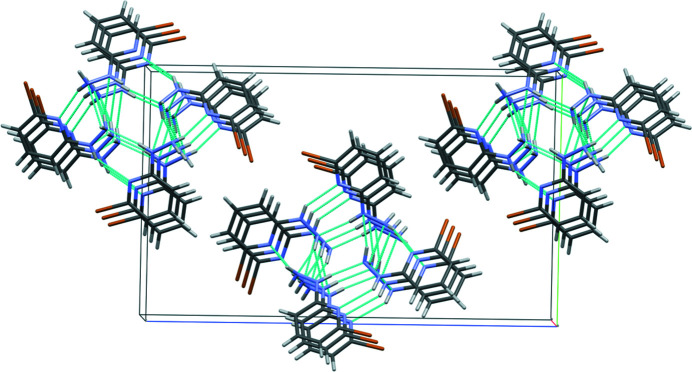
Mol­ecular packing of (**I**). Hydrogen bonds are shown as cyan dotted lines. Crystallographic axes colour codes: *a* – red; *b* – green; *c* – blue.

The conventional hydrogen bonding in the crystal structure of (**I**) is extensive and involves all nitro­gen atoms of both hydrazine groups and pyridine rings (Table 1 ▸) and is shown in Fig. 2 ▸. The hydrogen-bonding pattern is represented by a network of infinite chains, which propagate in the [100] direction. This network features 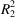 (7) rings, which are formed by almost coplanar mol­ecules (**I1**) and (**I2**), as shown in Fig. 1 ▸, and which represent the shortest inter­molecular heteroatom contacts in the crystal. A centrepiece of the network is N3, which participates in five short heteroatom contacts, once as an acceptor and four times as a donor of hydrogen bonds [two bifurcated N—H⋯(N,N) links]. Over half the hydrogen-bonding contacts are multicentered and include two bifurcated hydrogen bonds for donor atoms H3*A* and H3*B*, and N6 acts as a double acceptor (Fig. 2 ▸; Table 1 ▸).

**Figure 3:**
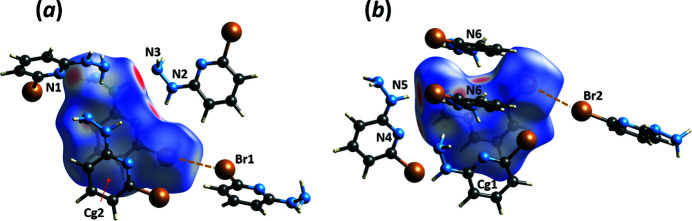
Views of the Hirshfeld surface for (*a*) mol­ecule (**I2**) and (*b*) mol­ecule (**I1**), mapped over *d*_norm_ in the range −0.56 to 0.97 a.u. with the blue-to-red color palette reflecting distances from a point on the surface to the closest nuclei. The neighboring mol­ecules involved in the shortest N—H⋯N hydrogen bonds, the Br⋯Br halogen bond, and the *Cg*⋯*Cg* stacking inter­actions are shown.

In addition, there is one short inter­molecular contact, Br1⋯Br2 [3.6328 (7) Å], which satisfies the distance and directionality conditions (Table 2 ▸) for a halogen bond (Desiraju *et al.*, 2013 ▸), with Br2 serving as a donor and Br1 as an acceptor of the bond, as shown in Fig. 3 ▸. Inter­molecular non-polar inter­actions, which may contribute to the stability of mol­ecular packing in the crystal, are represented by hydrogen–carbon contacts between the aromatic rings; the shortest of these contacts, C6—H6*A*⋯C9 [H6*A*⋯C9^i^ = 2.72 (3) Å, symmetry code: (i) −1 + *x*, *y*, *z*] is about 0.18 Å shorter than the sum of the van der Waals radii. The aromatic rings of both (**I1**) and (**I2**) are involved in a well-defined system of staggered π–π stacking inter­actions (Table 3 ▸). These various inter­actions can be seen in the Hirshfeld surface of the title compound (Fig. 3 ▸).

## Synthesis and crystallization

The title compound was prepared following an established synthetic route (Zoppellaro *et al.*, 2004 ▸). Specifically, 8.0 g (34 mmoles) of 2,6-di­bromo­pyridine, 15 ml (310 mmoles) of hydrazine hydrate, and 2 ml of 1-propanol were heated at 80°C for 12 h. The reaction mixture slowly separated into two layers, with the lower layer taking about 5 ml, then the mixture homogenized back. After cooling overnight at 4°C, the solution deposited pale-yellow needles of the title compound suitable for further X-ray diffraction studies.

## Refinement

Crystal data, data collection and structure refinement details are summarized in Table 4 ▸. Enanti­opurity of the crystal chosen for data collection was established on the basis of the Flack absolute structure parameter determined [0.012 (5) for 999 quotients (Parsons *et al.*, 2013 ▸)].

## Supplementary Material

Crystal structure: contains datablock(s) I. DOI: 10.1107/S2414314623001694/hb4424sup1.cif

Structure factors: contains datablock(s) I. DOI: 10.1107/S2414314623001694/hb4424Isup2.hkl

Supporting information file. DOI: 10.1107/S2414314623001694/hb4424Isup3.cml

CCDC reference: 2243041

Additional supporting information:  crystallographic information; 3D view; checkCIF report

## Figures and Tables

**Table 1:** Hydrogen-bond geometry (Å, °)

*D*—H⋯*A*	*D*—H	H⋯*A*	*D*⋯*A*	*D*—H⋯*A*
N2—H2*A*⋯N4	0.80 (3)	2.36 (3)	3.058 (3)	146 (3)
N3—H3*A*⋯N6^i^	0.85 (3)	2.43 (3)	3.212 (3)	154 (3)
N3—H3*A*⋯N5^ii^	0.85 (3)	2.67 (3)	3.149 (3)	117 (3)
N3—H3*B*⋯N2^iii^	0.84 (4)	2.74 (4)	3.543 (3)	161 (3)
N3—H3*B*⋯N6^ii^	0.84 (4)	2.69 (3)	3.183 (3)	119 (2)
N5—H5⋯N3	0.86 (3)	2.06 (3)	2.913 (3)	173 (3)
N6—H6*B*⋯N1^i^	0.82 (3)	2.46 (3)	3.257 (3)	164 (3)

Symmetry codes: (i) 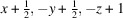 ; (ii) 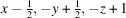 ; (iii) 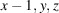 .

**Table 2:** Halogen-bond geometry (Å, °)

C—*D*⋯*A*—C	*D*⋯*A*	C—*D*⋯*A*	*D*⋯*A*—C	Symmetry code
C6—Br2⋯Br1—C1	3.6328 (7)	169.39 (6)	103.45 (7)	−*x* + 1, *y* + 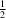 , −*z* + 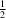

**Table 3:** π–π stacking geometry (Å, °) (*a*) perpendicular distance of *Cg*(*I*) on ring *J*; (*b*) perpendicular distance of *Cg*(*J*) on ring *I*; (*c*) dihedral angle between Planes *I* and *J*; (*d*) angle between *Cg*(*I*)–>*Cg*(*J*) vector and normal to plane *I*; (*e*) angle between *Cg*(*I*)–>*Cg*(*J*) vector and normal to plane *J*.

*Cg*(*I*)⋯*Cg*(*J*)	*Cg*i-*Cg*j	*Cg*(*I*)-perp *^*a*^*	*Cg*(*J*)-perp *^*b*^*	α*^*ac*^*	β*^*ad*^*	γ*^*e*^*	Slippage
*Cg*1⋯*Cg*1^iv^	3.9607 (14)	3.4889 (10)	–3.4890 (10)	0.03 (11)	28.2	28.2	1.875
*Cg*1⋯*Cg*1^v^	3.9605 (14)	–3.4889 (10)	3.4888 (10)	0.03 (11)	28.2	28.2	1.875
*Cg*2⋯*Cg*2^iv^	3.9607 (14)	3.4345 (9)	–3.4346 (9)	0.00 (11)	29.9	29.9	1.972
*Cg*2⋯*Cg*2^v^	3.9605 (14)	–3.4345 (9)	3.4345 (9)	0.00 (11)	29.9	29.9	1.972

Symmetry codes: (iv) *x* − 1, *y*, *z*; (v) *x* + 1, *y*, *z*.

**Table 4:** Experimental details

Crystal data	
Chemical formula	C_5_H_6_BrN_3_
*M*_r_	188.04
Crystal system, space group	Orthorhombic, *P*2_1_2_1_2_1_
Temperature (K)	150
*a*, *b*, *c* (Å)	3.9606 (3), 13.9649 (9), 23.0332 (14)
*V* (Å^3^)	1273.95 (15)
*Z*	8
Radiation type	Cu *K*α
μ (mm^−1^)	8.02
Crystal size (mm)	0.24 × 0.04 × 0.03

Data collection	
Diffractometer	Bruker APEXII area detector
Absorption correction	Multi-scan (*AXScale*; Bruker, 2021 ▸)
*T*_min_, *T*_max_	0.521, 0.754
No. of measured, independent and observed [*I* > 2σ(*I*)] reflections	25559, 2564, 2546
*R*_int_	0.027
(sin θ/λ)_max_ (Å^−1^)	0.624

Refinement	
*R*[*F* ^2^ > 2σ(*F* ^2^)], *wR*(*F* ^2^), *S*	0.013, 0.033, 1.07
No. of reflections	2564
No. of parameters	181
H-atom treatment	H atoms treated by a mixture of independent and constrained refinement
Δρ_max_, Δρ_min_ (e Å^−3^)	0.27, −0.36
Absolute structure	Flack *x* determined using 999 quotients [(*I* ^+^)−(*I* ^−^)]/[(*I* ^+^)+(*I* ^−^)] (Parsons *et al.*, 2013 ▸)
Absolute structure parameter	0.012 (5)

Computer programs: *APEX3* and *SAINT* (Bruker, 2021 ▸), *SHELXS* (Sheldrick, 2008 ▸), *SHELXL* (Sheldrick, 2015 ▸), *Crystal Explorer 17.5* (Mackenzie *et al.*, 2017 ▸), *Mercury* (Macrae *et al.*, 2020 ▸), *OLEX2* (Dolomanov *et al.*, 2009 ▸), and *publCIF* (Westrip, 2010 ▸).

## full crystallographic data

### Crystal data

**Table:**

C_5_H_6_BrN_3_	*D*_x_ = 1.961 Mg m^−^^3^
*M**_r_* = 188.04	Cu *K*α radiation, λ = 1.54178 Å
Orthorhombic, *P*2_1_2_1_2_1_	Cell parameters from 9416 reflections
*a* = 3.9606 (3) Å	θ = 3.7–73.7°
*b* = 13.9649 (9) Å	µ = 8.02 mm^−^^1^
*c* = 23.0332 (14) Å	*T* = 150 K
*V* = 1273.95 (15) Å^3^	Needle, colourless
*Z* = 8	0.24 × 0.04 × 0.03 mm
*F*(000) = 736	

### Data collection

**Table:**

Bruker APEXII area detector diffractometer	2564 independent reflections
Radiation source: Incoatec IMuS microfocus Cu tube	2546 reflections with *I* > 2σ(*I*)
Multi-layer optics monochromator	*R*_int_ = 0.027
φ and ω scans	θ_max_ = 74.3°, θ_min_ = 3.7°
Absorption correction: multi-scan (*AXScale*; Bruker, 2021)	*h* = −4→4
*T*_min_ = 0.521, *T*_max_ = 0.754	*k* = −17→17
25559 measured reflections	*l* = −28→28

### Refinement

**Table:**

Refinement on *F*^2^	Hydrogen site location: mixed
Least-squares matrix: full	H atoms treated by a mixture of independent and constrained refinement
*R*[*F*^2^ > 2σ(*F*^2^)] = 0.013	*w* = 1/[σ^2^(*F*_o_^2^) + (0.0151*P*)^2^ + 0.5435*P*] where *P* = (*F*_o_^2^ + 2*F*_c_^2^)/3
*wR*(*F*^2^) = 0.033	(Δ/σ)_max_ = 0.001
*S* = 1.07	Δρ_max_ = 0.27 e Å^−^^3^
2564 reflections	Δρ_min_ = −0.36 e Å^−^^3^
181 parameters	Absolute structure: Flack *x* determined using 999 quotients [(*I*^+^)-(*I*^-^)]/[(*I*^+^)+(*I*^-^)] (Parsons *et al.*, 2013)
0 restraints	Absolute structure parameter: 0.012 (5)
Primary atom site location: structure-invariant direct methods	

### Special details

**Table:**

Geometry. All esds (except the esd in the dihedral angle between two l.s. planes) are estimated using the full covariance matrix. The cell esds are taken into account individually in the estimation of esds in distances, angles and torsion angles; correlations between esds in cell parameters are only used when they are defined by crystal symmetry. An approximate (isotropic) treatment of cell esds is used for estimating esds involving l.s. planes.
Refinement. The hydrazine H atoms were treated by a mixture of independent and constrained refinement while the methine hydrogen atoms were initially placed in calculated positions. All hydrogen-atom coordinates were allowed to refine freely, while displacement parameters were constrained to ride on the carrier atoms [*U*_iso_(methine H) = 1.2*U*_eq_].

### Fractional atomic coordinates and isotropic or equivalent isotropic displacement parameters (Å^2^)

**Table:**

	*x*	*y*	*z*	*U*_iso_*/*U*_eq_	
Br2	0.82558 (6)	−0.11957 (2)	0.41033 (2)	0.02165 (7)	
N5	0.2752 (6)	0.10488 (14)	0.55037 (8)	0.0196 (4)	
N4	0.5314 (5)	−0.00629 (12)	0.49331 (8)	0.0161 (4)	
Br1	−0.03786 (6)	0.36607 (2)	0.24286 (2)	0.02079 (7)	
N2	0.2746 (6)	0.13007 (15)	0.39929 (8)	0.0252 (5)	
N1	0.1329 (5)	0.22862 (13)	0.32263 (8)	0.0150 (4)	
N6	0.1173 (5)	0.14093 (14)	0.60068 (8)	0.0185 (4)	
C3	0.4274 (6)	0.10092 (16)	0.24479 (10)	0.0209 (5)	
H3	0.526775	0.057251	0.218189	0.025*	
C8	0.6904 (6)	−0.12096 (17)	0.58795 (10)	0.0198 (4)	
H8	0.742112	−0.160750	0.620199	0.024*	
N3	0.1114 (6)	0.19152 (15)	0.43885 (9)	0.0198 (4)	
C9	0.5193 (6)	−0.03596 (15)	0.59669 (10)	0.0171 (4)	
H9	0.455892	−0.016115	0.634609	0.021*	
C5	0.2734 (6)	0.14643 (15)	0.34125 (9)	0.0171 (4)	
C1	0.1477 (6)	0.24529 (14)	0.26623 (10)	0.0153 (4)	
C4	0.4229 (6)	0.08000 (16)	0.30289 (10)	0.0194 (5)	
H4	0.518306	0.022107	0.317038	0.023*	
C2	0.2855 (7)	0.18666 (17)	0.22446 (10)	0.0198 (5)	
H2	0.284680	0.203175	0.184451	0.024*	
C6	0.6988 (6)	−0.08743 (15)	0.48825 (10)	0.0160 (4)	
C10	0.4416 (6)	0.02050 (15)	0.54774 (9)	0.0154 (4)	
C7	0.7876 (6)	−0.14892 (16)	0.53255 (10)	0.0194 (5)	
H7	0.907529	−0.206701	0.525612	0.023*	
H5	0.217 (8)	0.134 (2)	0.5193 (13)	0.023*	
H6A	−0.002 (8)	0.099 (2)	0.6194 (14)	0.029*	
H3A	0.189 (9)	0.247 (2)	0.4323 (14)	0.029*	
H6B	0.249 (9)	0.164 (2)	0.6242 (14)	0.029*	
H2A	0.340 (8)	0.080 (2)	0.4114 (13)	0.023*	
H3B	−0.094 (10)	0.192 (2)	0.4303 (14)	0.029*	

### Atomic displacement parameters (Å^2^)

**Table:**

	*U*^11^	*U*^22^	*U*^33^	*U*^12^	*U*^13^	*U*^23^
Br2	0.02599 (13)	0.02054 (11)	0.01841 (11)	0.00436 (10)	0.00240 (9)	−0.00398 (9)
N5	0.0309 (11)	0.0166 (9)	0.0112 (8)	0.0065 (8)	−0.0005 (8)	−0.0007 (7)
N4	0.0197 (10)	0.0138 (8)	0.0149 (8)	−0.0018 (8)	−0.0008 (8)	−0.0004 (6)
Br1	0.02350 (12)	0.02039 (11)	0.01847 (11)	0.00379 (9)	0.00074 (9)	0.00649 (8)
N2	0.0451 (14)	0.0163 (9)	0.0143 (9)	0.0125 (10)	0.0005 (9)	0.0026 (8)
N1	0.0174 (9)	0.0136 (8)	0.0138 (8)	−0.0005 (7)	−0.0022 (8)	−0.0009 (7)
N6	0.0234 (10)	0.0186 (9)	0.0136 (8)	0.0000 (8)	0.0019 (8)	−0.0032 (7)
C3	0.0215 (11)	0.0202 (10)	0.0209 (11)	0.0004 (9)	0.0017 (10)	−0.0061 (8)
C8	0.0198 (10)	0.0191 (10)	0.0205 (10)	−0.0022 (10)	−0.0067 (9)	0.0048 (9)
N3	0.0271 (12)	0.0169 (9)	0.0154 (9)	0.0034 (8)	−0.0009 (8)	0.0001 (7)
C9	0.0193 (11)	0.0183 (10)	0.0138 (9)	−0.0035 (9)	−0.0016 (9)	0.0005 (8)
C5	0.0205 (11)	0.0130 (10)	0.0177 (10)	−0.0023 (8)	−0.0040 (8)	−0.0006 (8)
C1	0.0146 (10)	0.0146 (9)	0.0165 (10)	−0.0007 (8)	−0.0020 (9)	0.0020 (8)
C4	0.0218 (12)	0.0139 (9)	0.0223 (11)	−0.0004 (8)	−0.0007 (9)	−0.0017 (8)
C2	0.0214 (12)	0.0232 (11)	0.0146 (10)	−0.0016 (9)	0.0003 (9)	−0.0005 (8)
C6	0.0163 (10)	0.0160 (10)	0.0157 (10)	−0.0016 (8)	0.0006 (9)	−0.0037 (8)
C10	0.0165 (10)	0.0142 (9)	0.0156 (10)	−0.0027 (8)	−0.0016 (9)	−0.0010 (8)
C7	0.0189 (11)	0.0141 (10)	0.0252 (11)	0.0005 (9)	−0.0027 (9)	0.0006 (8)

### Geometric parameters (Å, º)

**Table:**

Br2—C6	1.917 (2)	C5—C4	1.411 (3)
N5—N6	1.410 (3)	C1—C2	1.376 (3)
N5—C10	1.351 (3)	C6—C7	1.379 (3)
N4—C6	1.318 (3)	N2—H2A	0.80 (3)
N4—C10	1.356 (3)	N3—H3A	0.85 (3)
Br1—C1	1.917 (2)	N3—H3B	0.84 (4)
N2—N3	1.409 (3)	N5—H5	0.86 (3)
N2—C5	1.356 (3)	N6—H6A	0.87 (3)
N1—C5	1.346 (3)	N6—H6B	0.82 (3)
N1—C1	1.321 (3)	C2—H2	0.95
C3—C4	1.370 (3)	C3—H3	0.95
C3—C2	1.403 (3)	C4—H4	0.95
C8—C9	1.382 (3)	C7—H7	0.95
C8—C7	1.389 (3)	C8—H8	0.95
C9—C10	1.410 (3)	C9—H9	0.95
			
C10—N5—N6	124.36 (19)	N3—N2—H2A	117 (2)
C6—N4—C10	116.81 (19)	C5—N2—H2A	120 (2)
C5—N2—N3	122.2 (2)	N2—N3—H3A	106 (2)
C1—N1—C5	116.46 (19)	N2—N3—H3B	107 (2)
C4—C3—C2	120.2 (2)	H3A—N3—H3B	108 (3)
C9—C8—C7	120.8 (2)	N6—N5—H5	114 (2)
C8—C9—C10	118.1 (2)	C10—N5—H5	121 (2)
N2—C5—C4	120.3 (2)	N5—N6—H6A	114 (2)
N1—C5—N2	117.3 (2)	N5—N6—H6B	114 (2)
N1—C5—C4	122.3 (2)	H6A—N6—H6B	107 (3)
N1—C1—Br1	114.48 (16)	C1—C2—H2	122
N1—C1—C2	126.9 (2)	C3—C2—H2	122
C2—C1—Br1	118.63 (17)	C2—C3—H3	120
C3—C4—C5	118.5 (2)	C4—C3—H3	120
C1—C2—C3	115.7 (2)	C3—C4—H4	121
N4—C6—Br2	114.58 (16)	C5—C4—H4	121
N4—C6—C7	126.7 (2)	C6—C7—H7	122
C7—C6—Br2	118.69 (17)	C8—C7—H7	122
N5—C10—N4	114.20 (19)	C7—C8—H8	120
N5—C10—C9	123.9 (2)	C9—C8—H8	120
N4—C10—C9	121.9 (2)	C8—C9—H9	121
C6—C7—C8	115.7 (2)	C10—C9—H9	121
			
C5—N1—C1—Br1	−177.25 (16)	C10—N4—C6—Br2	−178.79 (16)
C5—N1—C1—C2	1.4 (4)	C10—N4—C6—C7	1.2 (4)
C1—N1—C5—N2	177.1 (2)	C6—N4—C10—N5	179.1 (2)
C1—N1—C5—C4	−1.3 (3)	C6—N4—C10—C9	−0.7 (3)
N3—N2—C5—N1	5.4 (3)	N6—N5—C10—N4	171.0 (2)
N3—N2—C5—C4	−176.2 (2)	N6—N5—C10—C9	−9.2 (4)
Br1—C1—C2—C3	177.84 (18)	Br2—C6—C7—C8	179.49 (17)
N1—C1—C2—C3	−0.8 (4)	N4—C6—C7—C8	−0.5 (4)
C1—C2—C3—C4	0.0 (4)	C6—C7—C8—C9	−0.7 (3)
C2—C3—C4—C5	0.0 (4)	C7—C8—C9—C10	1.1 (3)
C3—C4—C5—N1	0.7 (4)	C8—C9—C10—N4	−0.3 (3)
C3—C4—C5—N2	−177.6 (2)	C8—C9—C10—N5	179.9 (2)
C5—N2—N3—H3A	−54 (2)	C10—N5—N6—H6A	−45 (2)
C5—N2—N3—H3B	61 (2)	C10—N5—N6—H6B	77 (2)
H2A—N2—N3—H3A	137 (3)	H5—N5—N6—H6A	121 (3)
H2A—N2—N3—H3B	−109 (3)	H5—N5—N6—H6B	−117 (3)
H2A—N2—C5—N1	174 (3)	H5—N5—C10—N4	6 (2)
H2A—N2—C5—C4	−7 (3)	H5—N5—C10—C9	−174 (2)
Br1—C1—C2—H2	−2	Br2—C6—C7—H7	−1
N1—C1—C2—H2	179	N4—C6—C7—H7	180
C1—C2—C3—H3	−180	C6—C7—C8—H8	179
H2—C2—C3—C4	−180	H7—C7—C8—C9	179
H2—C2—C3—H3	0	H7—C7—C8—H8	−1
C2—C3—C4—H4	180	C7—C8—C9—H9	−179
H3—C3—C4—C5	180	H8—C8—C9—C10	−179
H3—C3—C4—H4	0	H8—C8—C9—H9	1
H4—C4—C5—N1	−179	H9—C9—C10—N4	180
C1—N1—C9—H9B	176	H9—C9—C10—N5	0
H4—C4—C5—N2	2		

### Hydrogen-bond geometry (Å, º)

**Table:**

*D*—H···*A*	*D*—H	H···*A*	*D*···*A*	*D*—H···*A*
N2—H2*A*···N4	0.80 (3)	2.36 (3)	3.058 (3)	146 (3)
N3—H3*A*···N6^i^	0.85 (3)	2.43 (3)	3.212 (3)	154 (3)
N3—H3*A*···N5^ii^	0.85 (3)	2.67 (3)	3.149 (3)	117 (3)
N3—H3*B*···N2^iii^	0.84 (4)	2.74 (4)	3.543 (3)	161 (3)
N3—H3*B*···N6^ii^	0.84 (4)	2.69 (3)	3.183 (3)	119 (2)
N5—H5···N3	0.86 (3)	2.06 (3)	2.913 (3)	173 (3)
N6—H6*B*···N1^i^	0.82 (3)	2.46 (3)	3.257 (3)	164 (3)

Symmetry codes: (i) *x*+1/2, −*y*+1/2, −*z*+1; (ii) *x*−1/2, −*y*+1/2, −*z*+1; (iii) *x*−1, *y*, *z*.
